# Real-Time Feedback Training to Improve Postural Control in a Patient With Femoral Neck Fracture and Severe Dementia: A Case Study

**DOI:** 10.7759/cureus.85050

**Published:** 2025-05-29

**Authors:** Taiga Okada, Taizan Shirakawa

**Affiliations:** 1 Rehabilitation, Matterhorn Rehabilitation Hospital, Hiroshima, JPN; 2 Orthopedics, Matterhorn Rehabilitation Hospital, Hiroshima, JPN

**Keywords:** balance adjustment system, cop control, elderly patient, femoral neck fracture, real-time body sway reduction, severe dementia, static balance training

## Abstract

Older adults with Alzheimer’s disease have a higher risk of falling compared to cognitively healthy individuals, partly due to the combined presence of cognitive decline and motor impairments. In older adults with severe dementia, conventional balance training is often difficult to implement because of reduced comprehension and difficulty following complex instructions. To address these challenges, there is increasing interest in balance interventions that are cognitively accessible, feasible under static conditions, and do not rely heavily on active motor learning or verbal instruction. Balance training using the Balance Adjustment System (BASYS) (Tec Gihan Co., Ltd., Kyoto, Japan) can be performed in a static standing position and is expected to offer clinical benefits. This case study examined the changes observed following BASYS-based balance training, which reduces body sway in real-time, on balance improvement. The subject was a 97-year-old female patient with severe Alzheimer’s disease and a femoral neck fracture. Conventional balance training was used initially, followed by an intervention phase of balance training with BASYS. The observed changes following the intervention were assessed based on changes in functional balance and center of pressure (COP) parameters during static standing. Improvements in both functional balance and COP parameters were observed during the period in which balance training with BASYS was implemented. Furthermore, spectral analysis of the anterior-posterior (AP) component of the COP revealed a reduction in the relative power of the mid- and low-frequency bands of the AP spectral density, which may suggest potential alterations in balance control mechanisms. These findings suggest that a static standing approach could support balance training in patients with severe dementia.

## Introduction

Older adults with Alzheimer’s disease (AD) are known to have an elevated risk of falling compared to older adults without dementia. Recent meta-analyses have reported that the annual fall prevalence among people with AD ranges from approximately 44% to 45%, substantially higher than the 26.5% reported in the general older adult population [1,2]. Additionally, rates of recurrent and injury-related falls are also elevated in this group, with up to 42% experiencing repeated falls and nearly half sustaining injuries due to falls [1]. A study on individuals with mild to moderate Alzheimer’s disease found that their standing balance was significantly impaired under various static and dynamic conditions [3]. Furthermore, in patients with severe dementia, the interplay between motor and cognitive decline necessitates careful consideration when designing and implementing assessment and intervention strategies [4]. However, research on longitudinal changes in postural control and the effectiveness of interventions for patients with severe dementia remains limited.

Given these challenges, it is imperative to develop balanced training interventions that accommodate the limitations inherent in severe dementia. There is currently no clear consensus regarding effective training strategies for individuals with severe cognitive impairment [5]. Due to poor verbal comprehension and difficulties in task execution, individuals with severe cognitive impairment often struggle to participate in conventional balance training, particularly those that rely on complex or multistep instructions. In addition, increased postural sway during quiet standing has been associated with an elevated risk of falls in individuals with dementia [6]. Therefore, there is a need for simpler and more accessible intervention approaches that specifically target static standing balance in this population.

In recent years, the Balance Adjustment System (BASYS) (Tec Gihan Co., Ltd., Kyoto, Japan) has been developed, and it may offer a potential solution to address these challenges. This device facilitates coordination between voluntary movements and reflects adjustments in an individual’s standing posture adjustment. Furthermore, BASYS can modulate an individual’s body sway in real-time, either reducing it (in-phase mode) or amplifying it (anti-phase mode). Notably, the in-phase mode differs from conventional balance training methods, offering a novel interventional approach. Although the effects of BASYS-based training have not been studied in healthy adults or older individuals, nor has its use for real-time sway reduction during static standing been examined, related research has shown improvements in functional performance in stroke patients [7] and reduced postural sway in young adults with chronic ankle instability [8]. These findings suggest the potential utility of real-time feedback-based balance training in various populations, including those with neurological or musculoskeletal impairments.

Despite its potential, balance training with real-time feedback to reduce body sway in very elderly patients with severe dementia remains largely unexplored. In this context, real-time feedback refers to subtle movements of the support surface (BASYS), which continuously adjusts in response to the patient’s center of pressure (COP) shifts during quiet standing. The platform’s motion is limited to within 15% of the COP displacement and is not consciously perceived by the patient. This design allows the system to gently guide the body’s balance responses and support postural control without requiring active cognitive involvement. Since complex balance exercises are often impractical for this population, interventions that can be performed in a static standing position may provide significant clinical benefits. The aim of this case report is to describe changes in functional balance and COP stability following balance training with real-time feedback using BASYS in an elderly patient with a femoral neck fracture and severe dementia. Importantly, we also analyze the relative power of the anterior-posterior (AP) component of the spectral density (AP-PSD), providing insight into balance control mechanisms and stability.

## Case presentation

**Figure 1:**
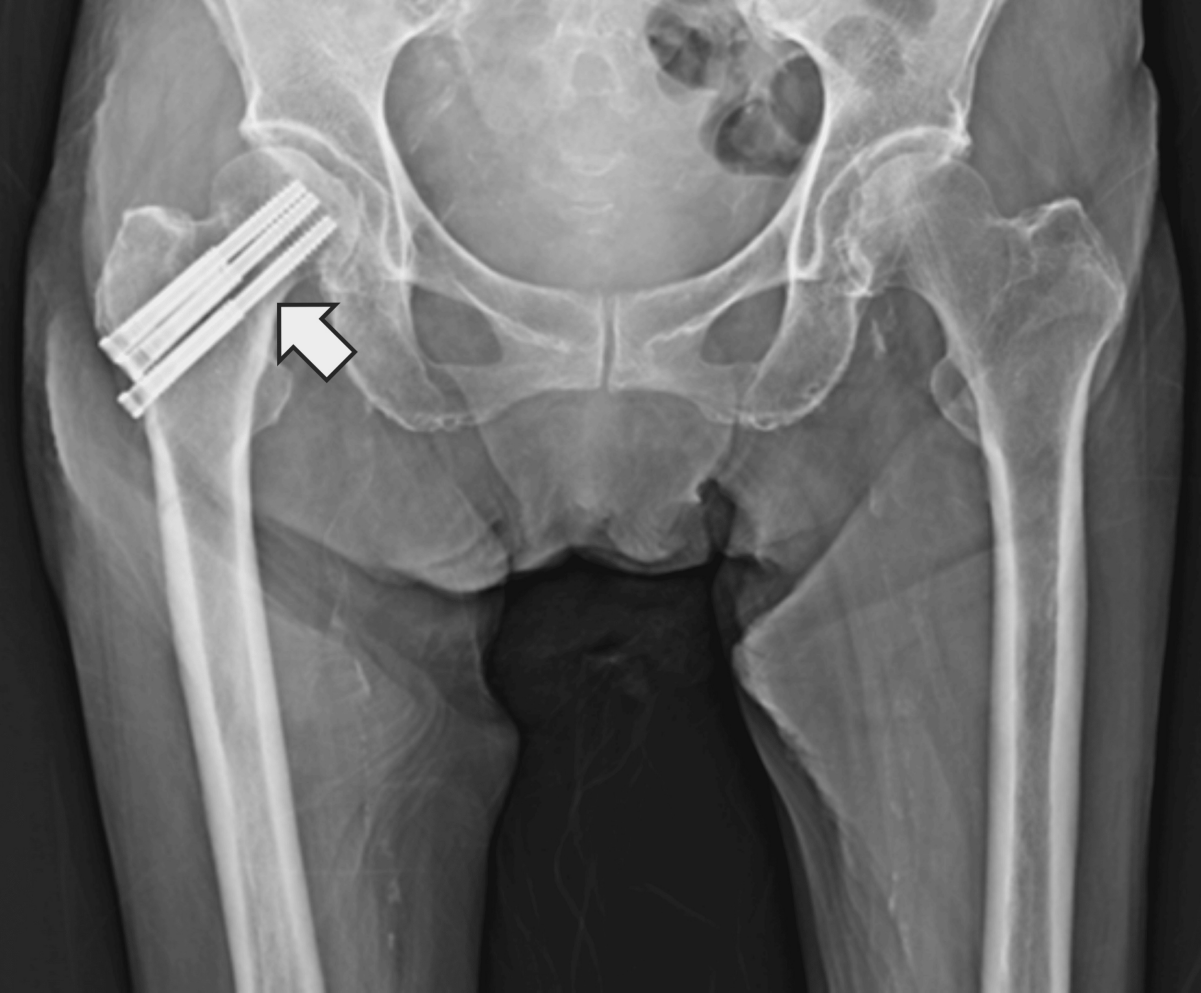
Post-operative image This image shows the right hip joint after open reduction and internal fixation (ORIF). The arrow indicates the site of ORIF.

A 97-year-old female patient sustained a femoral neck fracture following a fall at her residential facility. Prior to the injury, she was able to ambulate independently indoors and used a cane for outdoor mobility. She was independent in transfers, toileting, and basic activities of daily living, although she required assistance for bathing through care services. The diagnosis was established at an acute care hospital via diagnostic imaging, and she subsequently underwent open reduction and internal fixation (Figure 1).

After a 19-day acute hospital stay for post-operative care, the patient was admitted to our hospital for an intensive rehabilitation program that included physical therapy and occupational therapy. The patient’s clinical characteristics upon hospital admission to our hospital are summarized in Table 1. She exhibited severe cognitive impairment, as evidenced by a Mini-Mental State Examination (MMSE) total score of 6 at admission. The subscale scores indicated partial preservation of certain cognitive abilities, including 1 point for orientation to time (season), 2 points for immediate recall, 1 point for delayed recall, 1 point for naming, and 1 point for following oral commands. With the aid of simplified verbal instructions and imitation support, she was partially able to understand and respond to commands and was able to complete the training under continuous supervision. Given these preserved abilities, we judged that real-time feedback-based static balance training using BASYS could be feasibly implemented in this case.

**Table 1:** Clinical characteristics of the patient BBS: Berg balance scale. FRT: Functional reach test. MMT: Manual muscle testing. A1: start of phase A. B1: Start of phase B. B2: After one week of starting phase B. B3: After two weeks of starting phase B

Category	Item	A1	B1	B2	B3
BBS score	Total score	26	24	29	34
	Sitting to standing	3	3	3	3
	Standing unsupported	3	3	3	3
	Sitting unsupported	4	4	4	4
	Standing to sitting	3	3	4	3
	Transfers	2	2	2	2
	Standing with eyes closed	3	3	3	3
	Standing with feet together	1	1	1	3
	Reaching forward with outstretched arm while standing	2	2	2	2
	Retrieving object from floor	3	0	3	3
	Turning to look behind over left and right shoulders while standing	1	0	2	2
	Turning 360 degrees	0	2	2	2
	Placing alternate foot on stool while standing unsupported	1	1	0	1
	Standing with one foot in front (tandem stance)	0	0	0	2
	Standing on one leg	0	0	0	1
FRT (cm)	Right	11.3	7.0	11.0	10.5
	Left	15.4	11.5	11.0	21.5
MMT	Hip flexion - Right	2	2	2	2
	Hip flexion - Left	3	3	3	3
	Hip extension - Right	2	2	2	2
	Hip extension - Left	2	2	2	2
	Hip abduction - Right	2	2	2	2
	Hip abduction - Left	2	2	2	2
	Knee extension - Right	3	3	3	3
	Knee extension - Left	3	3	3	3

The trial had an AB design. In this case study, the intervention and observation periods were defined in advance, although the study design was not registered in any database. An AB design was chosen as the patient had severe dementia, making a within-subject comparison more appropriate for assessing the intervention's effects. In phase A (days 10-24 post-admission), conventional physical therapy was provided, consisting of gait training, balance training, and activities of daily living (ADL) training. During phase B (days 25-38 post-admission), the conventional balance training was replaced by a real-time postural feedback-based intervention using the balance adjustment system, BASYS (MPF-5050B; Tec Gihan Co., Ltd., Kyoto, Japan), while gait and ADL training continued unchanged. For descriptive purposes, assessments were conducted at four-time points: A1 (start of phase A), B1 (start of phase B), B2 (seven days after starting phase B), and B3 (14 days after starting phase B).

In phase B, the real-time postural feedback-based intervention was conducted using the in-phase mode of the device. The real-time feedback was provided through subtle floor movement beneath the feet. In the in-phase mode, the platform moved in real-time in the same direction as the patient's COP displacement in the anterior-posterior direction, thereby reducing body sway along that axis. The mechanical feedback operated below the level of conscious awareness, with the platform movement scaled to 15% of the sensed COP displacement. The in-phase condition was selected based on the patient's postural characteristics evaluated during phase A. Clinical observation during phase A revealed episodes of pronounced body sway during static standing, which were visibly recognizable without the need for instrumental measurement. In addition, the anterior-posterior power spectral density (AP-PSD) analysis indicated that the majority of the power was concentrated in the low- and mid-frequency bands, with minimal contribution from the high-frequency band. This distribution suggested a pattern of unstable postural control characterized by slow and large sway. Based on these observations, the in-phase training mode was selected to suppress excessive sway and promote postural stability, with the primary aim of addressing the patient's unstable postural control.

**Figure 2:**
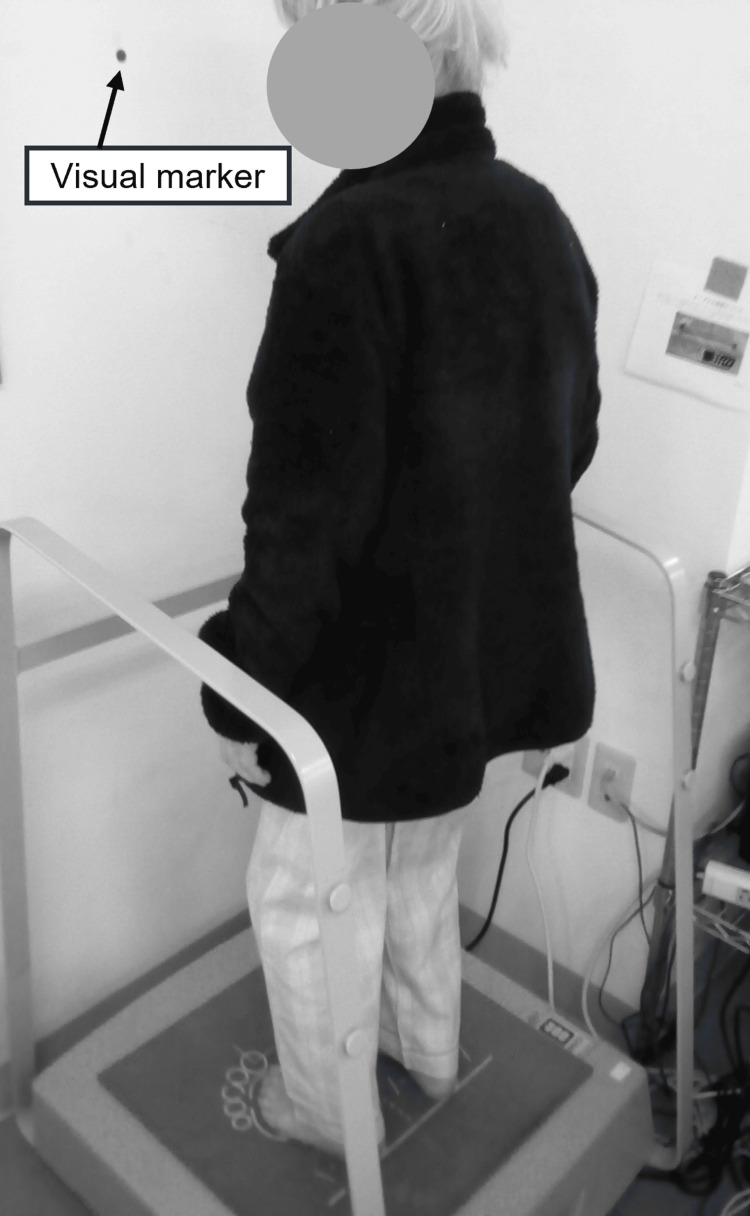
Patient positioning for the balance training intervention on BASYS The patient focused on a visual marker to minimize head and neck movement. Verbal instructions were provided to maintain an upright posture. The marker was placed on the wall at the patient’s eye level to facilitate consistent gaze direction and postural alignment.

To ensure proper execution, verbal instructions were given at the beginning of each session, directing the patient to “stand straight and avoid swaying.” The patient performed this intervention for three sets of one minute per day. Considering the patient’s fatigue, a rest period of 2 to 3 minutes was given between each set. During each session, the patient stood in a static upright posture with eyes open, positioning the feet according to predefined foot placement guidelines. A visual marker was positioned on the wall at the patient’s eye level to promote a natural upright posture and facilitate consistent visual engagement. The patient was instructed to focus on the marker to minimize head and neck movements (Figure 2). Observable deviations in head or eye orientation were used as an indicator of gaze direction. When such deviations were noted during training or measurement, verbal prompts were provided to redirect focus. If gaze deviations were observed during measurement, the assessment was repeated to ensure consistency. Repetition occurred once each in the B1 and B2 phases due to gaze deviation. Despite occasional deviations during the intervention sessions, the patient was able to continue training with verbal redirection without interruption. The device detected the user’s COP in real time using embedded force sensors beneath the support surface. The COP data were processed through an internal algorithm, which drove a motorized platform to move in the same direction as the detected sway. All the procedures were approved by the ethics committee of the Matterhorn Rehabilitation Hospital, Hiroshima, Japan (ethics review number: MRH24002), and conformed to the tenets of the Declaration of Helsinki. This study conforms to all CAse REport (CARE) guidelines and reports the required information accordingly.

Evaluation and data analysis

To evaluate balance, the patient’s performance was assessed using the Berg Balance Scale (BBS), the Functional Reach Test (FRT), and objective postural sway measurements obtained via BASYS. Each BASYS assessment lasted 60 seconds, and the following outcomes were derived from the COP data: total trajectory length, 95% ellipse area, and the relative power of the anterior-posterior (AP) component of the spectral density (AP-PSD). To accommodate the patient’s cognitive limitations, the BBS and FRT were administered using simplified verbal instructions and visual demonstrations. Assessments were conducted at four-time points: A1 (start of Phase A), B1 (start of Phase B), B2 (7 days after B1), and B3 (14 days after B1).

Data were filtered using a low-pass digital filter with a cutoff frequency of 10 Hz, applied through the software included with BASYS. The total trajectory length of the COP represents the path length of the center of pressure, based on data recorded from variations in foot pressure. Additionally, the 95% ellipse area represents the area of an ellipse that approximates the COP trajectory. AP-PSD was calculated for three frequency bands: low-frequency (0-0.3 Hz), mid-frequency (0.3-1 Hz), and high-frequency (1-3 Hz).

Results of the intervention

Clinical Evaluation

Table 1 summarizes the results of balance assessments conducted at four-time points. The BBS score was 26 at phase A1, 24 at B1, 29 at B2, and 34 at B3.

The Progression of Static Standing Balance

**Figure 3:**
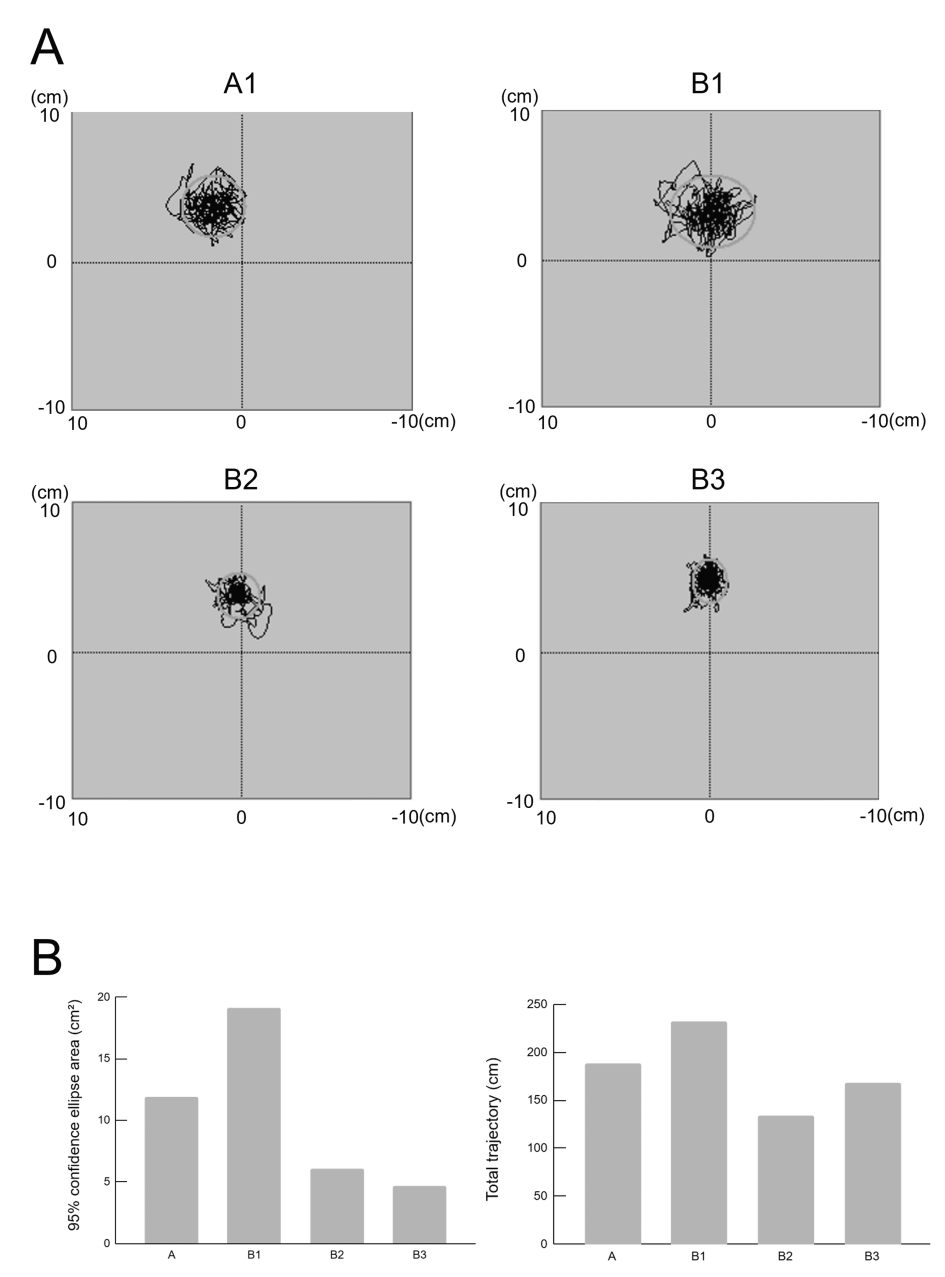
Transition of the center of pressure (COP) parameter across each phase of static standing balance. (A) COP trajectories during quiet standing in each phase. Gray circles indicate the areas of the 95% confidence ellipses. (B) Changes in 95% confidence ellipse area and total trajectory length. A1: start of phase A. B1: start of phase B. B2: after one week of starting phase B. B3: after two weeks of starting phase B. The image is created by the author.

In the COP analysis, the 95% confidence ellipse area was 11.881 cm² at phase A1, 19.093 cm² at B1, 6.058 cm² at B2, and 4.711 cm² at B3. The total trajectory length was 188.057 mm at phase A1, 232.145 mm at B1, 133.642 mm at B2, and 168.060 mm at B3 (Figure 3).

**Figure 4:**
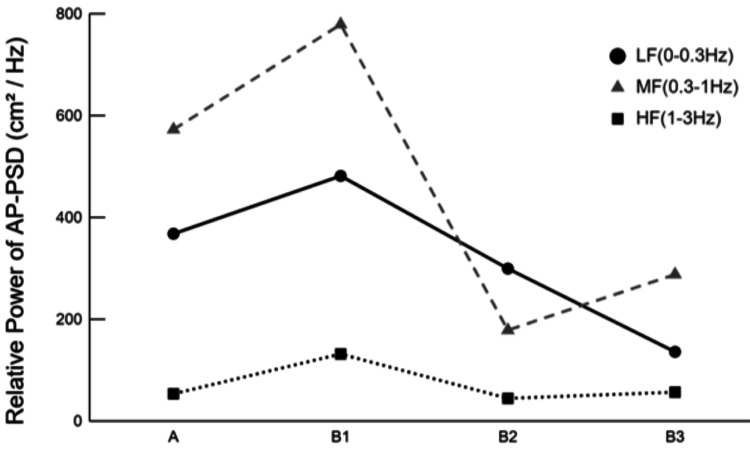
Changes in relative power of AP-PSD AP-PSD: Anterior-posterior power spectral density. LF: Low-frequency. MF: Mid-frequency. HF: High-frequency. A1: Start of phase A. B1: Start of phase B. B2: After one week of starting phase B. B3: After two weeks of starting phase B. The image is created by the author.

For the AP-PSD, the low-frequency power was 368.956 at phase A1, 482.640 at B1, 300.494 at B2, and 137.012 at B3. The mid-frequency power was 573.920 at phase A1, 780.330 at B1, 179.581 at B2, and 289.105 at B3. The high-frequency power was 54.548 at phase A1, 132.769 at B1, 45.618 at B2, and 57.765 at B3 (Figure 4).

Effect of In-Phase Mode on Postural Sway

**Figure 5:**
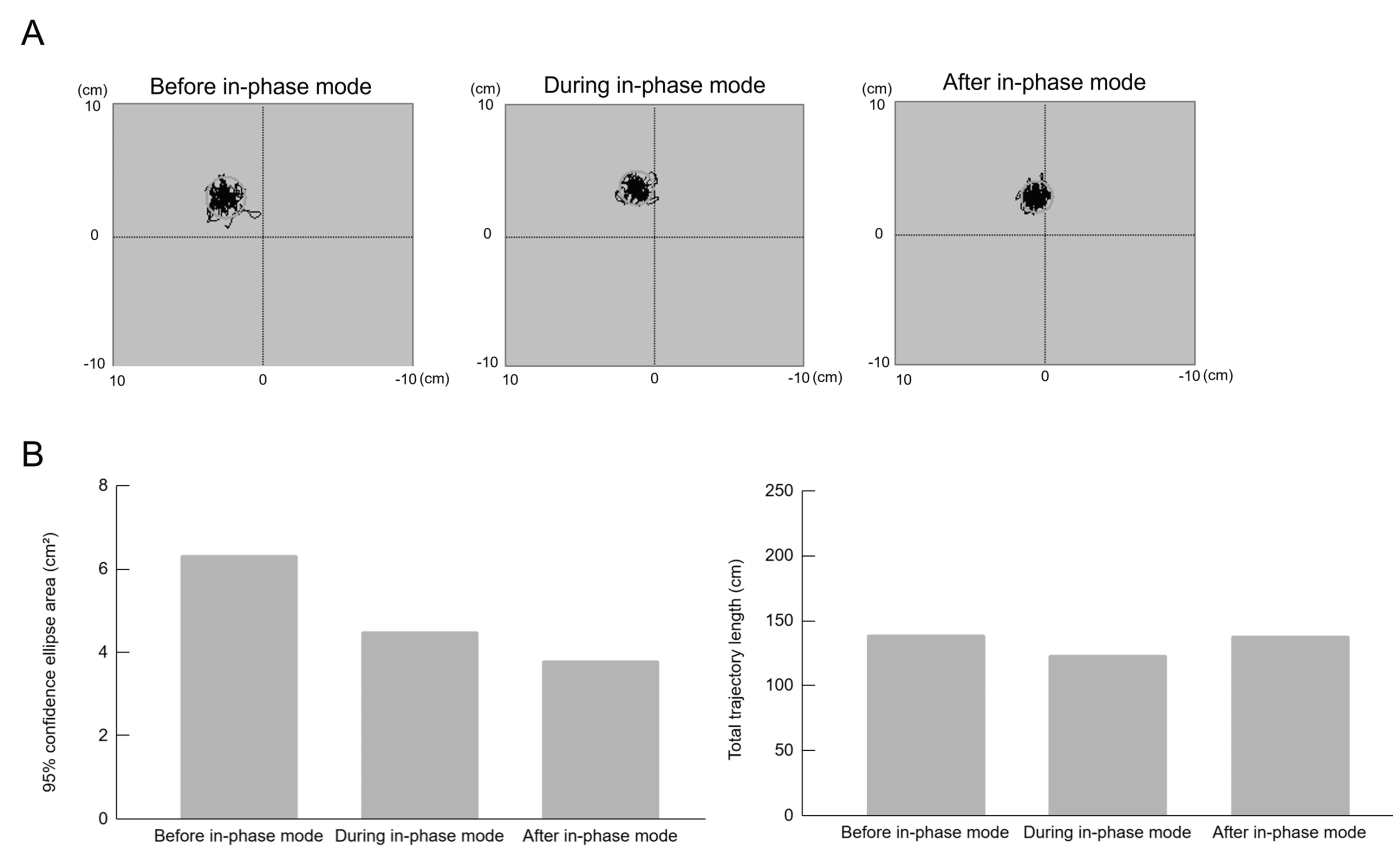
Representative example of COP trajectories and parameters obtained before, during, and after in-phase mode balance training on day three of phase B The image is created by the author. (A) Center of pressure (COP) trajectories during quiet standing before, during, and after the in-phase mode on the third day of phase B. Gray circles indicate the areas of the 95% confidence ellipses. (B) Changes in the area of the 95% confidence ellipse and total trajectory length before, during, and after the in-phase mode on the third day of phase B.

In the COP analysis, the 95% confidence ellipse area was 6.339 cm² before, 4.492 cm² during, and 3.812 cm² after the in-phase mode. Similarly, the total trajectory length was 139.472 cm before, 123.888 cm during, and 138.945 cm after the in-phase mode (Figure 5). These measurements were obtained on the third day of phase B and are presented as a representative example of the observed changes during in-phase mode.

## Discussion

The real-time postural feedback intervention using BASYS was associated with increases in Berg Balance Scale (BBS) scores and reductions in the 95% confidence ellipse area of the COP. Additionally, the relative power of the AP-PSD showed decreases in both the mid- and low-frequency bands.

Given that conventional balance training typically requires a relatively high level of cognitive engagement, its effectiveness may be limited in patients with severe dementia [5,9]. In contrast, an intervention that reduces body sway in real time may support postural control through implicit feedback mechanisms, with minimal reliance on active cognitive processing. As long as the patient is able to maintain a standing position, the intervention can be applied without requiring complex task execution or explicit instructions. Accordingly, this type of intervention may represent a feasible and practical rehabilitation option for improving balance and reducing fall risk in cognitively impaired elderly patients. These findings may therefore indicate that such an approach could help address some of the challenges faced by individuals with significant cognitive impairment, for whom traditional rehabilitation methods often fall short [10].

The patient in this case exhibited severe cognitive impairment; however, the observed improvements in postural stability may have been partially supported by unconscious sensorimotor processes. Theoretical models of postural control suggest that upright stance is regulated by feedback mechanisms that integrate proprioceptive, vestibular, and visual inputs and that these processes involve internal representations and coordination strategies that can function with minimal conscious involvement [11,12]. In this context, the subtle mechanical perturbations delivered through the BASYS platform may have stimulated peripheral sensory receptors, such as muscle spindles, thereby facilitating automatic postural adjustments without the need for cognitive processing [13]. Although these mechanisms were not directly evaluated in the present study, they may provide a useful explanation for the balance improvements observed in a patient with substantial cognitive limitations.

Regarding the course of BBS scores, a slight decline was observed from A1 (26) to B1 (24), which falls within the standard error of measurement (2.3 points) and may reflect natural variability in clinical performance rather than a true deterioration [14]. The score then increased from B1 (24) to B3 (34). Although this change appears large, there is currently no established minimum detectable change for individuals with severe cognitive impairment, making it difficult to determine the clinical significance of this finding.

Interestingly, although the intervention targeted quiet standing, improvements were observed in dynamic balance tasks on the BBS, such as turning and tandem standing. Generally, task specificity is a critical principle in motor learning [15], and transfer of skills between tasks tends to be limited, particularly in populations with cognitive impairments. In this regard, the influence of static postural training with BASYS on untrained dynamic tasks remains to be fully elucidated. Nevertheless, a pilot randomized controlled trial using the same real-time feedback system in patients with chronic stroke suggested that static standing balance training was associated with improvements in the Timed Up and Go test [7]. Although differences in patient characteristics, training frequency, and cognitive status preclude direct comparison, this finding raises the possibility that under certain conditions, improvements in static postural control may partially transfer to functional mobility tasks.

Further insight into the observed changes was provided by frequency-domain analysis. Notably, the intervention was associated with a reduction in sway within the low- and mid-frequency bands of the COP during quiet standing, as evidenced by the relative power analysis of the AP-PSD. These spectral changes were accompanied by a decrease in the 95% confidence ellipse area, suggesting an improvement in static postural stability. However, a slight increase in the total trajectory length was noted between B2 and B3. Several, non-exclusive interpretations may account for this finding. One possibility is the adoption of a postural stiffening strategy, whereby large amplitude sway is suppressed at the expense of increased small, high-frequency corrections [16]. Another explanation may be a shift in postural regulation strategies, as the concurrent increase in mid-frequency power could reflect enhanced reliance on vestibular and somatosensory inputs for balance control [17-20]. Finally, day-to-day fluctuations inherent to clinical measurement cannot be entirely excluded. Taken together, while the intervention appears to have contributed to static stability, the observed pattern may also indicate adjustments in postural control strategies that warrant further investigation.

Several limitations of this study should be considered when interpreting the findings. First, the validity of balance assessments may be affected by the patient’s severe dementia, despite the use of simplified instructions and visual cues. Cognitive function was assessed using the MMSE, but more comprehensive evaluation tools may be needed to clarify the influence of cognitive deficits on balance. Second, the absence of a follow-up period limits conclusions about the durability of the observed effects. Third, although the intervention was delivered consistently over two weeks, the overall training volume was low. While dosage is an important factor in rehabilitation outcomes, no established guideline exists for optimal volume in real-time feedback-based balance training using BASYS. Further research is needed to address these issues.

## Conclusions

This intervention involved balance training with real-time feedback aimed at reducing body sway in a patient with a femoral neck fracture and severe dementia. Changes were observed in functional balance and postural stability, including reductions in body sway and power within the mid- and low-frequency bands of the COP. These findings suggest that postural control strategies during quiet standing may be influenced by real-time feedback. Further research is needed to confirm these findings and examine their potential application in balance rehabilitation for individuals with severe cognitive impairment.
